# Individual Variation and the Bilingual Advantage—Factors that Modulate the Effect of Bilingualism on Cognitive Control and Cognitive Reserve

**DOI:** 10.3390/bs9120120

**Published:** 2019-11-21

**Authors:** Maurits van den Noort, Esli Struys, Peggy Bosch

**Affiliations:** 1 Research Group of Pain and Neuroscience, Kyung Hee University, Seoul 130-701, Korea; 2 Brussels Institute for Applied Linguistics, Vrije Universiteit Brussel, 1050 Brussels, Belgium; Esli.Struys@vub.be; 3 Psychiatric Research Group, LVR-Klinik Bedburg-Hau, 47511 Bedburg-Hau, Germany; p.bosch@donders.ru.nl; 4 Donders Institute for Brain, Cognition and Behaviour, Radboud University, 6525 Nijmegen, The Netherlands

This editorial is an introduction to the special issue ‘Individual variation and the bilingual advantage—factors that modulate the effect of bilingualism on cognitive control’. It provides a brief overview of the research field, discusses the 13 main studies of the special issue, and gives some important directions for future research.

The number of bilingual and multilingual speakers is steadily growing in many parts of the world [1]. How do bilinguals manage two or more language systems in their daily interactions and how does being bilingual/multilingual affect brain functioning and vice versa? Previous research showed that cognitive control plays a key role during bilingual language management and in order to perform this task, brain areas closely related to cognitive control were found to be engaged [2]. The special role for cognitive control in this process is further supported by the fact that learning and using foreign languages were found to affect not only the expected linguistic domains, but surprisingly, also other non-linguistic domains, such as attention [3], inhibition [3], working memory [4], decision making [5] and, indeed, cognitive control [6]. Somehow learning languages (even at an early stage) seems to affect executive functioning [7] and brain structures [8]. In the literature, this phenomenon is referred to as the “bilingual advantage” [9], meaning that the bilingual’s use of two (or more) languages—selecting one, while inhibiting the other(s)—enhances executive control skills, which leads to an advantage in cognitive control skills in bilinguals compared to monolinguals [10].

The aim of this special issue is to provide an overview of studies published so far on bilingualism and cognitive control, as well as their findings, in an effort to determine whether or not a bilingual advantage in cognitive control really exists. Furthermore, the focus will be on individual, as well as methodological, factors such as socioeconomic status [11], immigrant status and ethnicity [12], cognitive capacity [13], culture [14], age [15], and experimental task used [15], all factors that might modulate the bilingual advantage in cognitive control. Finally, we will take a closer look at the cognitive reserve hypothesis [16] that states that individuals with more cognitive reserve have a reduced risk of suffering from brain diseases, such as dementia [17]. In addition to factors like a higher level of education [18], complex occupations [18], cognitively stimulating leisure activities [18], suggestions have been made that being bilingual/multilingual enhances the individual’s cognitive reserve [19]. Does the daily use of two or more languages protect the aging individual against cognitive decline [20]? Does lifelong bilingualism protect against brain diseases, such as dementia [21], later in life?

## 1. Bilingual Advantage in Cognitive Control

First, having an overview of the results to date on research on bilingual advantage in cognitive control is important. In order to do so, Van den Noort and colleagues [15] conducted a review study. They searched Medline, ScienceDirect, Scopus, and ERIC databases for all original data and reviewed studies on bilingualism and cognitive control, with a cut-off date of 31 October 2018. Please note that only studies involving healthy participants were included in this review; studies that were conducted on cognitive decline and brain disorders will be discussed at a later stage of this editorial. Their search resulted in 46 original studies and 10 review studies. The majority (54.3%) of the original studies, indeed, reported beneficial effects of bilingualism on cognitive control tasks. In 28.3% of the studies, mixed results were found whereas in 17.4% of the studies, evidence was found against the existence of a bilingual advantage in cognitive control. How can these mixed results be explained? The authors point to the large differences in the methodologies that were used in these studies. For instance, the selection of the bilingual participants varied widely (e.g., low proficiency versus high proficiency, young age versus older age, highly educated versus poorly educated second-language speakers, bilingual participants versus multilingual participants, etc.) over the studies, resulting in heterogeneous groups or incomparable studies. Secondly, most researchers used non-standardized tests to collect data. Due to missing norms, these results cannot be interpreted correctly. In future research, individual differences should be better accounted for, larger studies are needed (most studies so far used small samples), and the use of longitudinal designs is highly recommended because second language (L2) learning is a complex dynamic process. Nevertheless, the authors conclude that despite these limitations, some evidence was found for a bilingual advantage in cognitive control.

In the first original study of the present special issue, Fidler and Lochtman [22] were interested in whether or not cognate language processing (meaning the processing of words that have a common etymological origin [23]) affected cognitive control, resulting in a possible bilingual advantage. Their study focuses on the influence of Dutch-German cognates, respectively orthographic neighbors, on controlled language processing (i.e., response inhibition). Two versions of the Stroop task [24], one in Dutch and one in German, were performed by 30 native speakers of Dutch, of whom 15 spoke German as a foreign language and 15 did not. In addition, the Stroop task in German was performed by 15 French-speaking participants who spoke German as a foreign language. In the German Stroop task, additional advantages in congruent, as well as incongruent, trials were found for the two Dutch-speaking groups, which postulate the existence of a cognate-neighbor-facilitation effect and an orthographic-neighbor-facilitation effect, even when participants only know one of the two cognate languages. Interestingly, the results suggest the existence of a so-called “notification mechanism”, a mechanism in the bilingual brain that is activated when dealing with cognates and orthographic neighbors. However, further research on this notification mechanism is needed in order to gain insights into the mechanism’s underlying learning processes.

In the second original study conducted by Nour and colleagues [25], the authors used the Attention Network Test (ANT) [26] to investigate the relation between interpreting training and experience and attentional network components (e.g., alerting, orienting, and executive attention [27]). Previous research has shown bilinguals to outperform monolinguals in cognitive control [10]; however, do extremely proficient bilinguals, like professional interpreters, perform similarly? The researchers tested three groups: a group consisting of 17 interpreting students, a group consisting of 21 translation students, and a group consisting of 21 professional interpreters. A mixed design was used. The professional interpreters were tested only once while the interpreting and the translation student groups were tested longitudinally (at the beginning and the end of their Master’s program). The results showed different attention network dynamics for professional interpreters and interpreter students compared to translation students with respect to alertness and the executive network. First, interpreting students showed higher levels of alertness with a cost of reduced accuracy. Moreover, the alerting effect in interpreting students showed more resistance to training (meaning that interpreting training had less effect than translation training on alerting). Thirdly, interpreting students showed a larger alerting effect compared to professional interpreters while both younger student groups showed a smaller conflict effect than professional interpreters. In contrast, professional interpreters performed significantly better than both student groups in executive accuracy scores, confirming that they use a different responding strategy. In future research, the inclusion of a control group for professional interpreters is recommended by the researchers in order to be able to investigate the effect of long-term interpreting experience on the attention network. This study [25] makes clear that the level of L2 proficiency and the amount of daily use of the two languages seem to be important factors that affect executive functioning (including cognitive control).

In their original study, Wu and colleagues [28] investigated the effect of bilingualism on inhibition control in 93 Uyghur–Chinese bilingual young adults. Thirty-one participants were Uyghur first language (L1) dominant, 31 participants were Chinese L2 dominant, and 31 participants were Uyghur–Chinese balanced (meaning individuals had equal proficiency in both the native language and the L2). They were particularly interested in the effect of within bilingual factors (i.e., dominance types of Uyghur–Chinese bilinguals) on two experimental tasks: a Flanker task (which is a so-called “stimulus–stimulus” task) [29] and a Simon task (which is a so-called “stimulus–response” task) [30]. Moreover, they compared the bilinguals’ performance scores on both cognitive control tasks, regarding a possible trade-off between speed and accuracy. The results showed that the within-bilingual factor (i.e., language dominance type; in the present study meaning whether the participants were Uyghur (L1) dominant, were Chinese (L2) dominant or were Uyghur_Chinese balanced), had no explicit effect on the performance of cognitive control tasks and that the advantage of balanced bilinguals was not present in the separate analysis of speed and accuracy. A second main finding of their study was that regardless of the degrees of bilingual proficiency, the underlying mechanism of bilingual language inhibitory control depended, to a large extent, on the type of stimulus–stimulus conflict resolution that was present in both language recognition and production processes. Wu and colleagues [28] concluded that exposure to different sociolinguistic contexts where different types of inhibition are induced, such as stimulus–stimulus or stimulus–response conflict, may lead to various patterns in strategic task tendencies in bilingual cognitive processing.

Woumans and colleagues [31] investigated language-switching behavior in adults. Previous research showed that language-switching behavior was a determining factor for the bilingual advantage. In their study, a bilingual advantage in the executive functions of inhibition and shifting was hypothesized. Inhibition and shifting performances of monolingual and bilingual participants on a Simon task [30] and a color-shape switching task [32] were analyzed. Furthermore, the relation between these executive functions and language-switching proficiency was tested using a semantic verbal fluency task [33]; the individual’s self-estimated language-switching score and the actual language-switching score were analyzed using an adapted version of the verbal fluency task [31]. A bilingual advantage for shifting, but not for inhibition, was found; moreover, that advantage was not related to language-switching behavior. No relation between subjective and objective measures of switching abilities was found. These findings support the existence of a bilingual advantage. On the other hand, these findings validate the elusiveness of bilingual benefits, as demonstrated by the absence of bilingual benefits on the measure of inhibition. The results of the present study [31] add to the discussion on the validity of switching measures.

The fifth original article on the bilingual advantage in cognitive control was conducted by Boumeester and colleagues [34] who focused on late bi-/multilingualism (meaning that the foreign languages were acquired at or after the age of five). The impact of proficiency-based and amount-of-use-based degrees of multilingualism in different modalities (i.e., speaking, listening, writing, and reading) on inhibition, disengagement of attention, and switching were investigated in 54 late bi-/multilinguals. Their results [34] showed that only proficiency-based degrees of multilingualism affected cognitive abilities. In particular, a marginally significant independent positive effect of mean proficiency in foreign languages in the writing and the listening modalities on inhibition (in the literature known as a flanker effect [29]) was found, as was a significant negative effect of L2 proficiency in the listening modality on disengagement of attention (in the literature referred to as a sequential congruency effect [35]). The first conclusion that those authors drew was that their results seemed to suggest that only those speakers who had reached a certain proficiency threshold in more than one foreign language showed a bilingual advantage. Their second conclusion was that when the impact of proficiency-based degrees of multilingualism on cognitive abilities was considered, the listening and the writing modalities mattered.

In contrast to the five original studies on the relation between bilingualism and cognitive control in which adults were investigated [22,25,28,31,34], Haft and colleagues [36] investigated a group of young children. They were interested in the possible associations between bilingualism and cognitive flexibility—a relationship that has shown mixed findings in prior literature [37,38]. In addition, they explored relationships between bilingualism and attentional fluctuations, which represent consistency in attentional control and contribute to cognitive performance, a topic that has never been studied before. A sample of 120 kindergarten children was included in their study. Of those 120 children, 16 had no L2 exposure and 104 had some L2 exposure (including a subsample of 24 children with L2 exposure since birth). In line with previous research, in which null findings were found when confounding variables were adequately controlled and the experimental tasks were standardized [39], Haft and colleagues [36] expected to find no bilingual advantage in either cognitive flexibility or attentional fluctuations. Their results showed, indeed, no proof for the existence of a bilingual advantage in cognitive flexibility. Moreover, no evidence was found for an association between bilingualism and attentional fluctuations. Nevertheless, they stressed that despite the fact that they had found no support for a bilingual advantage in general cognition (and that this null-effect had also been reported in other recent studies on the bilingual experience [40,41]), these results should in no way discourage the development of dual-language proficiency and L2 learning because knowing a foreign language brings advantages outside of the cognitive domain, such as the option for understanding different cultures, broadening of the horizons, open-mindedness, and expanded communicative abilities.

In the seventh original article (and the second study investigating bilingual children), Festman and Schwieter [42] were interested in the topic of the individual’s self-concept. Cognitive representations and beliefs are what comprise an individual’s self-concept [43]. Previous research discovered that a positive and strong relation existed between a positive self-concept and academic achievement [44]. Festman and Schwieter were interested in the relationship between domain-specific self-concepts and standardized assessments of reading and writing competencies against the background of potential differences in self-concept between monolingual and multilingual children. They investigated 125 third-grade children who were enrolled in primary school in Germany: 69 monolingual children and 56 multilingual children. The results showed that while between-group comparisons revealed similar results for self-concept or reading competency between monolingual and multilingual children, monolingual children were found to be better than multilingual children in spelling. Moreover, the correlation analyses revealed significant positive correlations between domain-specific self-concepts and academic achievement in reading comprehension, reading fluency, and spelling in both the monolingual and the multilingual groups. Importantly, both the monolingual children and the multilingual children were able to estimate correctly their academic achievement (e.g., reading and spelling performances). The authors of the present study conclude that metacognition and executive functions can lead to better educational outcomes; however, they are of the opinion that more research with a larger multilingual sample, allowing for subgroup comparisons which were not possible in the study by Festman and Schwieter [42], is needed.

The original studies on the bilingual advantage in cognitive control in adults and children that have been discussed thus far have all used standard behavioral measurements (performance scores and reaction times). The study by Ouzia and colleagues [45] is unique because in addition to behavioral measures, the authors used eye-tracking [46]. In their study, they took a closer look at the role of emotions in cognitive control. The attentional control theory [47] is a theory that approaches the relationship between anxiety and executive function. That theory relies on the assumption that anxiety (including non-clinical levels) adversely affects processing efficiency (often measured through reaction times) to a greater extent than it affects accuracy (performance effectiveness) [48]. Those authors used eye tracking, as well as behavioral measures of inhibition, in 31 young and healthy monolingual and 27 highly proficient bilingual adults. Trait anxiety was found to be a reliable risk factor for decreased inhibitory control accuracy in bilingual, but not monolingual, participants. These findings, therefore, indicate that adverse emotional traits may differentially modulate performance in monolingual and bilingual individuals, an interpretation which has implications both for attentional control theory [47] and future research on bilingual cognition.

If progress in the field is to be made, a critical look at the research conducted so far is important. What lessons can we learn? How can the quality of the research on the bilingual advantage in cognitive control be further increased? In the opinion article by de Bruin [49], attention was drawn to the fact that all bilinguals differ from one another and that one cannot simply treat them as one homogenous group. Differences in bilingual experiences can affect language-related processes; moreover, findings in the literature suggest that bilingual experience modulates executive functioning as well. Within the field, we have seen in recent years an increased focus on individual differences (e.g., age of acquisition, as well as language proficiency, use, and switching) between bilinguals. Nevertheless, most studies do not assess these individual differences between bilinguals sufficiently. De Bruin [49] makes several important recommendations that certainly should help the bilingual-advantage research field to develop further: (1) More detailed descriptions of the bilingual participants in studies are needed, particularly for studies that aim to investigate the fine-grained effects of bilingual experiences on executive functioning; (2) the use of (standardized) objective proficiency measurements is strongly recommended. These assessments should be used for a more detailed description of the bilingual participants in the methods section of the paper. Moreover, they are important when studying the effects of bilingual experiences on executive functioning; (3) better validations based on actual recordings of language use in daily life should be conducted to assess the reliability of the currently available and future questionnaires and measurements. To conclude, careful examination and description of not only a bilingual’s proficiency and age of acquisition, but also their language use and switching, as well as the different interactional contexts in which they use their languages, are crucial for achieving a better understanding of the effects of bilingualism within and across studies.

Finally, as we have discussed, in the presented studies on the bilingual advantage in cognitive control, the debate on possible cognitive advantages bilinguals have over monolinguals continues to occupy the research community [37,38,39,40,41]. Moreover, an ever-growing body of research is focusing on adjudicating whether an effect on cognition exists [38,39] when using two or more languages regularly. In their opinion article, Poarch and Krott [50] stressed the importance of identifying attenuating, modulating, and confounding factors in research on the bilingual advantage in cognition. Importantly, at the same time, they argued for a change in perspective concerning what is deemed an advantage and what is not and argued for more ecologically valid research that investigates real-life advantages.

## 2. Cognitive Reserve Hypothesis

In the second, smaller part of our special issue, we focused on the cognitive reserve hypothesis [16]. Bilingualism has been put forward as a life experience that, similar to musical training [51] or being physically active [52], may boost cognitive performance [15] and slow age-related cognitive decline [53]. In the first study conducted by Van den Noort and colleagues [54], the literature is reviewed in order to provide an overview of the state-of-the-art results in the field. They searched Medline, ScienceDirect, Scopus, and ERIC databases for all original data and reviewed studies on bilingualism and the cognitive reserve hypothesis, with a cut-off date of 31 March 2019. Van den Noort and colleagues found 34 eligible studies. Mixed results were found with respect to the protective effect of bilingualism against cognitive decline. Several studies showed a protective effect whereas other studies failed to find it. Moreover, evidence for a delay in the onset of dementia of between 4 and 5.5 years in bilingual individuals compared to monolinguals was found in several studies, but not in all. Methodological differences in the set-ups of the studies seem to explain these mixed results. Lifelong bilingualism is a complex, individual process, and many factors seem to influence this and need to be further investigated.

The second study on the cognitive reserve hypothesis was conducted by Pot and colleagues [55], who focused on bilingualism in older adults while taking individual differences into account. Three sections in their paper respond to their three objectives: (1) The first section involved 387 older adults in the multilingual north of The Netherlands and focused on the question of how cognitive control is influenced by language control. More precisely, the intricate clustering of modulating individual factors as deterministic of cognitive outcomes of bilingual experiences at the older end of the lifespan was investigated; (2) the second section focused on older adults that turned bilingual later in life (i.e., through third-age language-learning programs). By relating cognitive, social, and linguistic outcomes of third-age language learning to those of lifelong bilingualism, a better understanding of the intricate relationship between language and cognitive control could be achieved. (3) In the third paper section, the first two were combined, resulting in a proposal for a flipped research perspective and a blueprint for work relating cognitive and social individual differences. Pot and colleagues [55] used the example of monolingual seniors and their baseline performance as predictors of foreign language learning success (i.e., rate and proficiency). Such proactive designs incorporating both behavioral and neural baseline data complement the reactive effect studies reviewed and discussed above to arrive ultimately at a better understanding of cognitive and language control and, eventually, of the protective effect of lifelong bilingualism/multilingualism.

## 3. Conclusions

This special issue perfectly illustrates the dynamics of this research field. Many international research groups are investigating intriguing hypotheses related to the bilingual advantage [9] and the cognitive reserve hypothesis [16]. On the other hand, this special issue also illustrates the difficulties of the field. Different researchers investigate different topics across the world. They study all kinds of monolingual, bilingual, and multilingual individuals with all kinds of experimental tasks, making comparisons of their results and interpretation of all of the results difficult and often impossible. This might explain why the results on the bilingual advantage in cognitive control [15] and the results on the cognitive reserve-enhancing effect of lifelong bilingualism and protection against dementia [54] are mixed.

How can we move forward? The present special issue tapped several topics that need to be addressed in future research on the bilingual advantage in cognitive control and on the relation between bilingualism and the cognitive reserve hypothesis. Firstly, individual differences should be better accounted for. Secondly, detailed descriptions of the bilingual participants are needed [49]. Thirdly, the use of (standardized) objective proficiency measurements is strongly recommended [49]. Moreover, larger study samples are needed [15]. So far, small study samples have been often used in research on the bilingual advantage in cognitive control and, to a lesser degree, in research on bilingualism and the cognitive reserve hypothesis. Furthermore, whether the bilingual advantage in cognitive control and the contribution to cognitive reserve are mainly limited to extremely proficient bilinguals that use both languages at a professional level the whole day, like interpreters, [25] and to multilingual individuals who have to switch and suppress languages extensively to a larger extent than bilinguals should be explicitly investigated [34]. Last, but not least, the use of longitudinal designs is highly recommended because L2 learning is a complex, dynamic process [15].

Lifelong bilingualism is a complex, individual process, and many factors seem to influence this and need to be further investigated using behavioral and neuroimaging measurements, but the intriguing research that has been conducted so far, as well as the studies that were presented in the present special issue, indicate the possible far-reaching consequences of lifelong bilingualism that seem to go beyond the linguistic domain [3,4,5,6]. Therefore, a change in perspective concerning what is deemed an advantage, and what is not, seems necessary [50], as does the need for more ecologically valid research that investigates real-life advantages [50]. In conclusion, we still have a long way to go, but little by little, we are making progress in understanding the underlying (brain) processes of lifelong bilingualism.
